# Where do T cell subsets stand in SARS-CoV-2 infection: an update

**DOI:** 10.3389/fcimb.2022.964265

**Published:** 2022-08-10

**Authors:** Mohammad Tarique, Mohd Suhail, Huma Naz, Naoshad Muhammad, Shams Tabrez, Torki A. Zughaibi, Adel M. Abuzenadah, Anwar M. Hashem, Hari Shankar, Chaman Saini, Alpana Sharma

**Affiliations:** ^1^ Department of Child Health, University of Missouri, Columbia, MO, United States; ^2^ King Fahd Medical Research Center, King Abdulaziz University, Jeddah, Saudi Arabia; ^3^ Department of Medical Laboratory Sciences, Faculty of Applied Medical Sciences, King Abdulaziz University, Jeddah, Saudi Arabia; ^4^ Department of Radiation Oncology, School of Medicine, Washington University in Saint Louis, Saint Louis, MO, United States; ^5^ Vaccines and Immunotherapy Unit, King Fahd Medical Research Center, King Abdulaziz University, Jeddah, Saudi Arabia; ^6^ India Council of Medical Research, New Delhi, India; ^7^ Department of Biochemistry, All India Institute of Medical Sciences (AIIMS), New Delhi, India

**Keywords:** COVID-19, immune response, T cell, immunological reaction, pathophysiology

## Abstract

An outbreak of coronavirus disease 2019 (COVID-19) emerged in China in December 2019 and spread so rapidly all around the globe. It’s continued and spreading more dangerously in India and Brazil with higher mortality rate. Understanding of the pathophysiology of COVID-19 depends on unraveling of interactional mechanism of SARS-CoV-2 and human immune response. The immune response is a complex process, which can be better understood by understanding the immunological response and pathological mechanisms of COVID-19, which will provide new treatments, increase treatment efficacy, and decrease mortality associated with the disease. In this review we present a amalgamate viewpoint based on the current available knowledge on COVID-19 which includes entry of the virus and multiplication of virus, its pathological effects on the cellular level, immunological reaction, systemic and organ presentation. T cells play a crucial role in controlling and clearing viral infections. Several studies have now shown that the severity of the COVID-19 disease is inversely correlated with the magnitude of the T cell response. Understanding SARS-CoV-2 T cell responses is of high interest because T cells are attractive vaccine targets and could help reduce COVID-19 severity. Even though there is a significant amount of literature regarding SARS-CoV-2, there are still very few studies focused on understanding the T cell response to this novel virus. Nevertheless, a majority of these studies focused on peripheral blood CD4+ and CD8+ T cells that were specific for viruses. The focus of this review is on different subtypes of T cell responses in COVID-19 patients, Th17, follicular helper T (TFH), regulatory T (Treg) cells, and less classical, invariant T cell populations, such as δγ T cells and mucosal-associated invariant T (MAIT) cells etc that could influence disease outcome.

## Introduction

There is ongoing outbreak of COVID-19, a novel coronavirus disease caused by SARS-CoV-2) emerged in China in December 2019 (Gates, 2020). The SARS-CoV-2 infection can manifest in a wide range of clinical manifestations, which can range from asymptomatic to mild COVID-19 infections, even severe COVID-19 infections which are hospital-acquired. When a patient is hospitalized, the likelihood of developing severe pneumonia, or the development of acute respiratory distress syndrome (ARDS), is very high (Yang et al., 2020). Presently, there are no effective antiviral drugs specifically designed to treat SARS-CoV-2 infection. Recent studies have provided important insights into the immune responses of patients who are hospitalized, even though relatively little is known about the immunology of asymptomatic or mild disease individuals (Oxenius and Zajac, 2022). According to current research, the adaptive immune system contributes a great deal to the progression of SARS-CoV-2 infection in a similar way as with any other respiratory viral infection (Bhardwaj et al., 2022). There is still some uncertainty regarding whether T-cell responses are useful or harmful in COVID-19, as well as whether they are suboptimal, dysfunctional, or excessive. There are studies that show evidence for both ends of the spectrum (de Candia et al., 2021; Luo et al., 2021; Toor et al., 2021).

In order to fully understand the pathogenesis of COVID-19, we need to know a pivotal role is played by the immune system. Both innate and adaptive immune responses need to be activated in a coordinated manner to eliminate the SARS-CoV-2 infection. The uncontrolled innate immune responses and compromised adaptive immune responses, resulting in widespread tissue destruction in COVID-19 infection. There are several clinically approved anti-viral drugs available for the treatment of COVID-19 infection, but they are not very promising. It is well known that T cells play an important role in controlling and preventing viral infections onset and spread (Bhardwaj et al., 2022). As a matter of fact, it has been demonstrated that COVID-19 disease severity inversely correlates with the magnitude of T cell response in several recent studies (Chen and John Wherry, 2020). In order to assist COVID-19 patients in decreasing the severity of their illness, it is of great interest to understand how T cells are responding to SARS-CoV-2 as they are attractive vaccine targets. There is a tremendous amount of literature that deals with SARS-CoV-2 specific research, however, studies specifically designed to understand how T cells are responding to this novel virus are relatively few in number. The majority of these studies are nonetheless focused on peripheral T cells that are specific for viruses as well as CD8+ T cells. The significance of other T cell subtypes should not be underestimated, however. For example, the role of follicular helper T (TFH), regulatory T (Treg) cells, Th17 as well as less classical, invariant T cell populations, such as the γδT cells and mucosal-associated invariant T cells (MAIT), may also be vital in the process, particularly in certain tissues such as the lung.

As a result of the emergence of SARS-CoV-2 variants that have the ability to evade antibodies, the first-generation COVID-19 vaccines have proven effective in mitigating severe illness and hospitalization, but recurring waves of infections lead to diminishing vaccine effectiveness. A study suggested that as a result of intestinal or parenteral immunization, viruses were controlled effectively and lung pathology was prevented, regardless of neutralizing antibodies present. When antibodies effectively neutralized the challenge virus, mucosal memory CD8 T cells provided little protection. When CD8 T cells were “unhelped” or not accompanied by CD4 T cells and neutralizing antibodies, mucosal memory CD8 T cells provided no protection against homologous SARSCoV-2. Nevertheless, memory CD4 and “helped” CD8 T cells in the lung, in the absence of detectable virus-neutralizing antibodies, provided significant protection against the antibody-resistant B1.351 (β) variant, without causing lung immunopathology (Kingstad-Bakke et al., 2022).

A comprehensive understanding of COVID-19 immunopathogenesis will contribute to the design of a satisfactory treatment for SARS-CoV-2 infection. The purpose of this review is to give a brief overview of the immunopathogenesis of COVID-19 infection, along with the conventional and unconventional T cell immune responses, which can be used to target the dysregulated immune response in COVID-19 infection.

## Pathogenesis and cytokine storm in COVID-19

**Figure 1:**
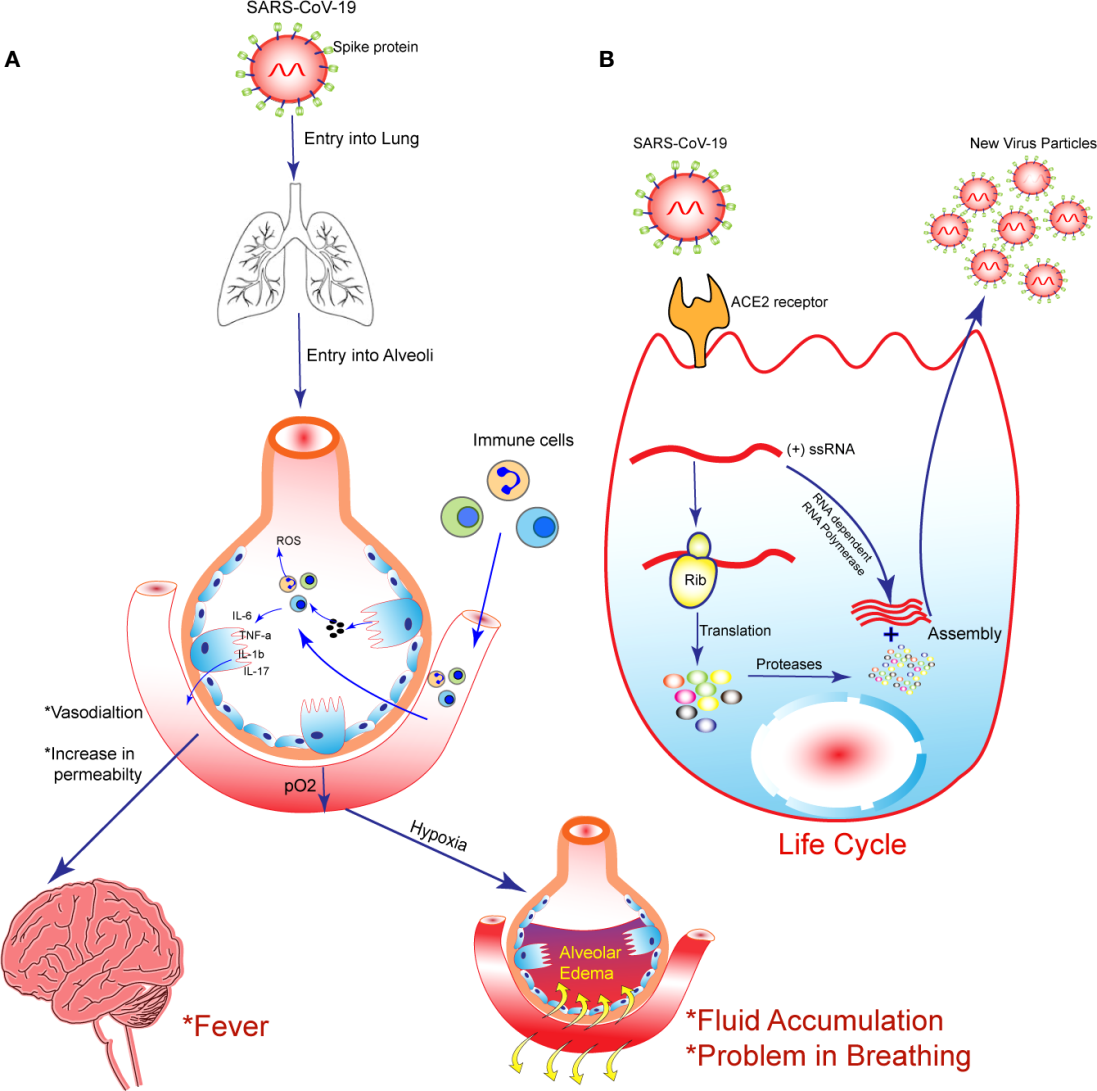
The following schematic diagram illustrates the steps taking place during the infection and replication of SARS-CoV-2 into host cells: **(A)** SARS-CoV-2 enter into the cell by binding to the ACE2 receptor present on the host cell membrane. As the virus replicates in the lung cells, it induces pyroptosis and the release of DAMPs (disease associated molecular patterns). Several pro-inflammatory cytokines and chemokines, including IL-6, IL-10, MIP1-α, MIP1-1β, and MCP-1, are secreted by adjoining epithelial cells, alveolar macrophages, and endothelial cells in response to contact with DAMPs. As a result of these cytokines and chemokines, monocytes, macrophages, and neutrophils are recruited to the lung, thereby releasing more inflammatory cytokines in the process, which cause hypoxia, fluid accumulation in lung, fever and acute lung injury **(B)** SARS-CoV-2 enters the cytoplasm and releases viral RNA, which is translated into polyproteins after entering the cytoplasm. The (+) stand genomic RNA is used as a template to begin replication transcription. Nucleoprotein complexes are formed from sub genomic RNA synthesized by transcription and the (+) strand genomic RNA. Virions are released from host cells following assembly of nucleoprotein complexes and proteins.

SARS-CoV-2, is a single strand virus, is transmitted from infected patients to healthy individuals *via* direct contact or by spreading respiratory droplets (Rothan and Byrareddy, 2020). COVID-19 symptoms that accompany the infection include fever, cough, pain, weakness, tightness in the chest, loss of taste and smell, as well as dyspnea, all of which are associated with acute respiratory distress syndrome (ARDS) (Huang et al., 2020). Hypoxemia, difficulty breathing, and onset of pulmonary edema are a few of the symptoms of ARDS, a condition that leads to respiratory failure by damaging lung endothelium and alveolar epithelium (Matthay et al., 2019). Both SARS-CoV2 and SARS-CoV1 are likely able to bind to the angiotensin converting enzyme 2 (ACE2) present in the lungs as well as other organs (Hoffmann et al., 2020). Amplification of the virus and its migration down through the conducting airways triggered a more robust innate immune response, which eventually spreads down the respiratory tract (**Figure 1**). As a result of viral infection, an immune response is triggered which occurs through the activation of innate immune cells that identify pathogen-associated molecule patterns. When the innate immune system fails to eliminate the virus, the adaptive immune system comes into play in order to fight the infection. When innate immune cells and adaptive immune cells are induced, their secretion of cytokines such as IL-6, IFN-γ, interferon-γ-induced protein-10 (IP-10) and monocyte chemoattractant protein-1 (MCP-1) begins. The cytokines and chemokines induce the influx of monocytes/macrophages and neutrophils at the site of infection from the bloodstream (Bhardwaj et al., 2022). Viral infection is cleared by the secretion of cytotoxic substances by these cells. It is normal for this response to be able to eliminate the virus, but sometimes the immune system is dysregulated, disrupting the balance of immunity (Perlman and Dandekar, 2005).

SARS-CoV-2 can be detectable through nasal swabs or sputum. The cytokine release syndrome (CRS) may lead to death among COVID-19-infected patients due to a substantial immune response characterized by abnormal cytokine production.” Several cytokines and chemokines participate in the “cytokine storm” in COVID-19 patients (Ragab et al., 2020), including IL-6, IL-1β, CXCL10, IL-2, IL-10, TNF-α and IFN-γ, but IL-6 plays an especially critical role, for its increased level in patients’ serum has been associated with respiratory failure, ARDS, and adverse clinical outcomes (Bhardwaj et al., 2022). This dysregulation of immune system and cytokine storm causes infiltration of neutrophils/monocytes, activation of T cells etc which cause accumulation of fluids in lungs, increases vascular permeability and leakage, increase c reactive proteins in liver and ultimately multi-system organ failure in COVID-19 patients. CRS plays a major role in the deterioration of COVID-19 patients, from pneumonia through acute respiratory distress syndrome (ARDS), cumulating in systemic inflammation. As a result of SARS-CoV-2 infection, dendritic cells are delayed in activation, resulting in impaired T-cell response (Zhou et al., 2020).

## Where does T cell stand to fight with SARS-CoV-2 infection?

Several variants of SARS-CoV-2 have been identified which reduce the ability of antibodies to block infection, posing concern that we will not be able to stop the disease. T cells are a critical aspect of the adaptive immune response. Although anti-viral antibodies can protect cells from infection, when antibody titer drop by the time after the infection or vaccination - some cells will ineluctably become infected. In such a scenario, T cells can help as cytotoxic T cells recognize infected cells and kill them. Major histocompatibility molecules on the plasma membrane present viral peptides to T cells so that they can detect infected cells and viral proteins may be used to make these T cell peptides. Conversely, only specific viral proteins, such as spike, can generate antibodies that block infection. In past years, the T cell response to SARS-CoV-2 infection has been well studied. The vaccine makers have dutifully included tests for neutralizing antibodies alongside the T cell responses in patients that some researchers have studied (Tea et al., 2021; Li et al., 2022). Although T cells are important for resolving most viral infections, they have never been included in the dialogue. They can help prevent and end disease through their ability to kill virus-infected cells.

**Figure 2:**
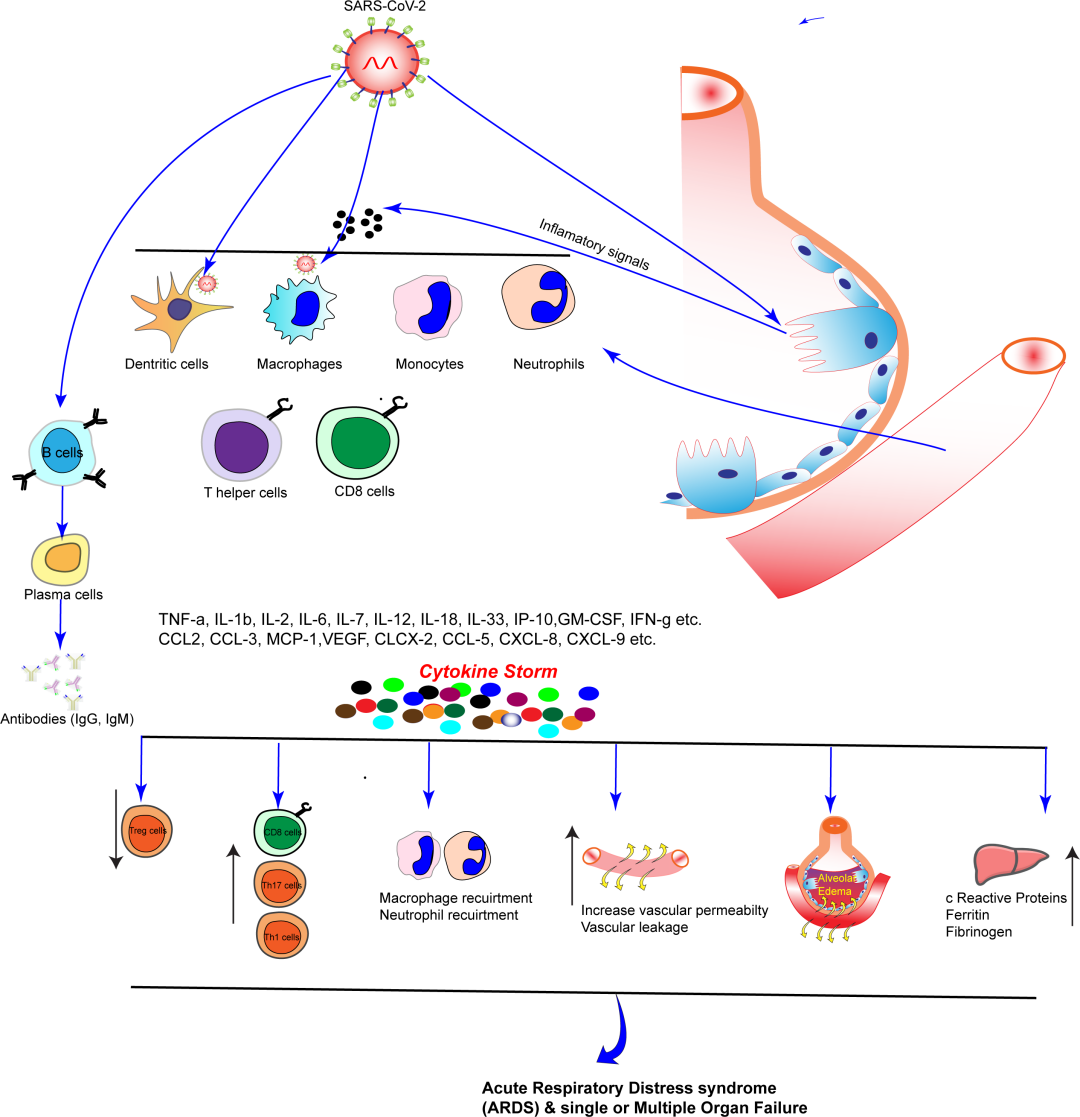
Recruitments of immune cells and damage of Lung epithelium and cytokine storm: SARS-CoV-2 enter into the cell by binding to the ACE2 receptor present on the host cell membrane. As the virus replicates in the lung cells and damage lung epithelium. Damaged lung epithelium cell secretes inflammatory signals and immune cells recruited in lung blood vessels which secrete pro-inflammatory cytokines and chemokines, including IL-6, IL-10, MIP1-α, MIP1-1β, and MCP-1, are secreted by adjoining epithelial cells, alveolar macrophages, and endothelial cells. As a result of these cytokines storm happens and increased blood vessels permeability, accumulation of fluid in the lung, hypoxia and ultimately acute respiratory distress syndrome (ARDS) and multiple organ failure.

The immune system relies on T cells to respond to viral infections, just as B cells produce antibodies (Swain et al., 2012). Epithelial cells in the airways are infected by the SARS-CoV-2 virus and it replicates within the cells, using the biochemical machinery of the host cells. Damage-associated molecules are being released as a result of programmed cell death of the host cell (Tay et al., 2020). When these molecules are recognized by macrophages and nearby endothelial and epithelial cells, they produce pro-inflammatory cytokines (IL-6, TNF-α, IL-1β, IFN-γ etc), such as chemokines (CXCL10, MCP-1, MIP-1α and 1β **Figures 1**, **2**). By triggering the release of chemokines and other cytokines, monocytes, macrophages, and T cells are recruited to the site of infection, leading to increased inflammation. This inflammation is triggered by the production of IFN-γ by recruited T cells (Bhardwaj et al., 2022). To understand disease pathogenesis, T cell immunity to SARS-CoV-2 must be characterized. However, few studies have examined T cell immunity and here we are discussing the advancement of T cell response to SARS-CoV-2.

CD4+ and CD8+ T cells in COVID-19 infection: Lymphopenia (a decrease in lymphocyte numbers), one of the symptoms of SARS-CoV-2 infection, is characteristic of severe infection. Several studies have suggested that disease intensity is correlated with lymphopenia, and it occurs more frequently in those with a higher mortality rate, particularly in severe cases (Tavakolpour et al., 2020). A pronounced decrease in CD4+ T cells, CD8+ T cells, and B cells has been reported in COVID-19 patients (Chen and John Wherry, 2020; Zhou et al., 2020). It is currently unknown how lymphopenia occurs in COVID-19, but it is known that most patients with severe disease have decreased T cell numbers, specifically CD8+ T cells, however the reason is unclear (Tavakolpour et al., 2020). Infections with other respiratory viruses, including influenza, have been associated with lymphopenia (McClain et al., 2013).

PD-1, a marker for exhausted T cells, appeared to be significantly more abundant in COVID-19 patients. As patients progressed from prodromal to overtly symptomatic stages, they expressed more PD-1 and Tim-3 on T cells (Diao et al., 2020). However, people with mild symptoms typically present with a slightly higher or normal T-cell count than those with moderate symptoms (Vabret et al., 2020). Even though peripheral T-cell loss in moderate to severe COVID-19, as well as other viral infections, is a phenomenon observed in other conditions, its cause remains elusive, and its cause is not yet certain, unlike MERS-CoV, where direct viral infection of T cells has been reported (Chu et al., 2016). According to recent research findings, the reduction in number of T cells in the blood may be a result of multiple mechanisms, including effects caused by inflammatory cytokines in the blood. IL-6, IL-10, and TNF-α seem to correlate with lymphopenia, while convalescent patients showed lower IL-6 and IL-10 levels, respectively (Diao et al., 2020; Liu et al., 2020). IFN-α and TNF-α can inhibit T cell recirculation, as they promote T cell adhesion to endothelial cells and retention in lymphoid tissues (Shiow et al., 2006). Chen et al. analyzed the spleens and hilar lymph nodes of COVID-19-suspected patients following autopsy and observed extensive lymphocyte death. They suggested that IL-6 and Fas-FasL interactions mediated apoptosis could be involved in the development of lymphocytopenia. It was found that tocilizumab, an IL-6 receptor antagonist, increases circulating lymphocytes in support of this hypothesis (Giamarellos-Bourboulis et al., 2020). Similarly, the presence of T cells in the peripheral circulation may be affected by their recruitment to infection sites. After death from ARDS brought about by SARS-CoV-2 infection, a post-mortem examination of the lung showed extensive lymphocyte infiltration further supporting this hypothesis (Xu et al., 2020).

SARS-CoV-2 infection hosts a CD4+ T cell response that can be impaired, activated excessively, or inappropriately and how this is corelated to disease outcome is an important question yet to be explored. When patients recover from COVID-19, they often exhibit strong and specific CD4+ T cell responses that are similar to what has been seen with influenza virus infection (Lee and Crane, 1988). Within days of onset of symptoms during acute COVID-19 infection, the frequency of SARS-CoV-2–specific CD4 T cells increase, leading to an increase in CD4 T cell activity (Sattler et al., 2020). There have been studies showing that some patients with severe COVID-19 have impaired CD4+ T cell function, which includes lowered IFN-γ production (Sattler et al., 2020). Other researchers believe the T cells have been over-activated (Mathew et al., 2020). A study suggested that patients recovered from SARS- CoV-2 had circulating virus-specific CD4+ T cells, which suggests T cell memory and immunity that may last a long time (Grifoni et al., 2020). There was also some evidence from some studies that CD8+ T cells from patients with COVID-19 had reduced cytokine production in response to *in vitro* stimulation from patients with severe COVID-19. Considering that some studies have reported signs of possibly exhausted T cells, other studies have shown an overly aggressive response by CD8+ T cells or high levels of activated CD8+ T cells with an increased cytotoxic response in patients with COVID-19 (Chen and John Wherry, 2020). Patients with mild disease had a higher proportion of CD8+ T cell responses, suggesting that CD8+ T cell responses may act as a protective factor (Peng et al., 2020). CD8+ T cell responses were predominantly directed against viral internal proteins rather than spike proteins, which would be an important element to consider when developing vaccines (Peng et al., 2020). A majority of SARS-CoV-2-recovering patients have CD8+ T cells that are specific for the virus (Grifoni et al., 2020). This indicates that there has been a highly specific CD8+ T cell response to the virus and that there is a long-lasting memory of these cells. Nevertheless, it remains to be seen whether these cells are sufficiently able to protect against future infections. It has been suggested that 40-60% of unexposed people have T cells that are reactive to SARS-CoV-2, a finding that supports the suggestion that circulating ‘common cold’ coronaviruses have shared similar recognition patterns with SARS-CoV-2 (Grifoni et al., 2020). An additional study reported cross-reactive memory T cells in patients who had recovered from SARS-CoV 17 years ago, as well as in individuals who had not suffered from SARS before (Le Bert et al., 2020). There have also been detectable SARS-CoV-2-specific memory T cells detected in seronegative healthy individuals who have been exposed to confirmed cases, suggesting an asymptomatic infection. The results of one study show that during the acute phase of the disease, SARS-CoV-2-specific T cells exhibited a high level of cytotoxicity, and a stem-like memory phenotype was observed in SARS-CoV-2-specific T cells in the convalescent phase (Sekine et al., 2020). Convalescent patients were found to have T cells specific for SARS-CoV-2 in most of the cases. This may be a sign that infection may trigger development of immunity to COVID-19 (Canete and Vinuesa, 2020).

GM-CSF-producing CD4+ T helper cells are identified as a distinct subset of CD4+ T cells. When activated, these T cells can recruit inflammatory myeloid cells to inflammation sites to enhance the immune response (Zhang et al., 2013). The presence of pathogenic CD4 cells that express GM-CSF and IL-6 is associated with the severity of COVID-19 disease. A higher number of CD4+ cells expressing GM-CSF and IL-6 was reported in COVID-19 patients admitted to intensive care units (Zhou et al., 2020). Zhao et al; observed that tissue-resident memory-like Th17 cells (Trm17 cells) remained in the lungs after the virus had been eradicated. Trm17 cells expressed CSF2 (GM-CSF) and IL17A, two potential pathogenic cytokines. The results of an interactome analysis suggest that Trm17 cells interact with lung macrophages and cytotoxic CD8+ T cells, which have been known to contribute to the severity of disease and the damage to the lungs. COVID-19 patients with higher serum levels of IL-17A and GM-CSF had more severe clinical symptoms (Zhao et al., 2021). Low GM-CSF levels are essential for long-term macrophage maintenance and alveolar macrophage development under homeostatic conditions. As COVID-19 progresses, increased GM-CSF levels may contribute to the activation of monocytes and macrophages as well as the induction of proinflammatory cytokines and contribute to the T cell-mediated acute pulmonary inflammation. Through increased GM-CSF levels in bronchoalveolar fluid, acute respiratory distress syndrome may be indirectly caused by a reduction in neutrophil apoptosis, which contributes to microvascular damage (Mu et al., 2021).

Follicular helper T (TFH) cells response in COVID-19: It is a special subset of CD4+ T cells that can help in enhancing the function of B cells by providing assistance through both cell-cell interactions as well as cytokine release, which helps improve the effectiveness of B cells resulting in antibodies being produced (Swain et al., 2012). The cells in Tfh express CXCR5 chemokine receptors, but they lack CCR7 chemokine receptors. These cells also expressed CD45RO, a transcriptional repressor, and a repressor called Bcl-6 (Crotty, 2014). A Tfh cell’s function is to act on specific cells within the immune system such as B-cells by recognizing CD40L and other costimulatory molecules, in addition to local cytokines like IL-21 (Elgueta et al., 2009). While the majority of Tfh cells reside in lymphoid GCs, circulating Tfh cells (cTfh) circulate in the bloodstream. It is estimated that about 10% of the total CD4 cell population in the bloodstream consists of cells expressing CXCR5, similar to the CXCR5 found in lymphoid tissue. Circulating T follicular helper cells (cTfh), defined as CXCR5+PD1+ CD4 T cells (Jandl et al., 2017). The activation of cTfh cells leads to the production of low levels of cytokines such as IFN-γ, IL-4, and IL-17 (Wang et al., 2016).

A certain subset of activated cTfh cells is seen in the bloodstream of those who are infected with SARS-CoV-2, or who have been vaccinated against it for a short period of time. The CD4 T-cells contribute to the immune response to antigens in vaccines or viruses (Cui et al., 2021). Therefore, activation of cTfh cells indicates a healthy and normal immune response to virus antigens. It is important to note, however, that different subsets of cTfh tend to be associated with different antibody production functions (Koutsakos et al., 2022). COVID-19 is characterized by the activation of a subset of cTfh cells with reduced CCR7 expression, high expression of PD-1 and high expression of ICOS-1 expression, which remains active for up to two weeks after symptoms occur (Gong et al., 2020). An acute infection usually results in the emergence of cTfh cells specific for the S protein, and these cells persist for six months or more (Salinas et al., 2021). A significant reduction in the number of cTFH cells that express CXCR3–CCR6+ was seen in the late stage of disease compared with the acute stage or early convalescence (Balachandran et al., 2022). Nonetheless, COVID-19 patients who have recovered have shown antigen-specific cTfh cells are able to produce IL-21 and IFN-γ. In acute COVID-19, the cTfh activation is used as a measure of the titer and avidity of the anti-RBD IgM antibodies (Juno et al., 2020). Similarly, high anti-anti-S antibody levels are observed in cTfh1 cells expressing CXCR3. As a matter of fact, the frequency of cTfh cells that are reactive with the SARS-CoV-2 S, N, and M antigens correlates with the number of neutralizing titers (Juno et al., 2020). There is some evidence that severe COVID-19 is associated with a reduction in the frequency of Tfh cells, with a higher frequency of cytotoxic cTfh cells expressing granzyme B and perforin (Kalfaoglu et al., 2021). It has been observed that current mRNA-based vaccines induce robust GC responses, which are accompanied by spike-specific activation of cTfh cells. It is known that these cells produce IFN-γ but not IL-17 while they peak at one month and then wane (Goel et al., 2021).

T regulatory cells in COVID-19: The regulatory T cells (Tregs) represent 5–10% of CD4+ T cells and express FoxP3 transcription factor and CD25, which play a critical role in regulating immune self-tolerance and various immune responses (Sakaguchi et al., 2020). The anti-inflammatory and tissue homeostasis properties of Treg cells go beyond suppressing autoimmune diseases (Sakaguchi et al., 2008; Saini et al., 2017). Various mechanisms are used by the Treg population to inhibit innate and adaptive immunity (Saini et al., 2018). These mechanisms include blocking antigen-presenting cell maturation *via* the CTLA-4 pathway, reducing IL-2 availability to conventional T cells with constitutive expression of the high-affinity IL-2 receptor, and secreting immunosuppressive molecules (IL-10, TGF-β, IL-35) (Tarique et al., 2017; Tarique et al., 2017). In the context of acute viral infection, medical research on Tregs in humans is limited. Studies have, however, suggested that Treg cells are also involved in viral infection immunopathology. These cells suppress immunity-mediated mechanisms of tissue damage by targeting immune-mediated mechanisms of tissue protection and suppressing antiviral T cell responses (Robertson and Hasenkrug, 2006; Li et al., 2008). Researchers have demonstrated that Tregs possess protective properties against virus-mediated tissue damage, including inflammation caused by herpes simplex virus, HCV-induced liver damage, and hepatitis C virus-induced cirrhosis (Claassen et al., 2010), as well as encephalitis caused by coronaviruses (Anghelina et al., 2009). In contrast, from experimental and clinical studies, it is evident that Tregs are central to the pathophysiology of some respiratory diseases like chronic obstructive pulmonary disease, asthma, idiopathic pulmonary fibrosis (IPF) (Krishnamoorthy et al., 2012; Loebbermann et al., 2012; Singh et al., 2019). However, inconsistent reports exist regarding the role of Treg cells in COVID-19 infection.

It is unclear how T-regs regulate the antigen-specific immune response to SARS Coronavirus 2 (SARS-CoV-2). According to some studies, the number of Treg cells in COVID-19 has increased, while others have revealed an unchanged or decreased number (Chen et al., 2020; Tan et al., 2020). The number of regulatory T cells (CD3+CD4+CD25+CD127low+), naïve regulatory T cells (CD45RA+CD3+CD4+CD25+CD127low+), as well as induced regulatory T cells (CD45RO+CD3+CD4+CD25+CD127low+) are decreased in the circulation of COVID-19 patients as compared to healthy controls (Qin et al., 2020). Furthermore, both mild and severe COVID-19 patients had significantly higher Treg cell frequencies in lymphocytes. Interestingly, Treg cells from patients with severe disease expressed a higher amount of CD25, further supporting increased immunoregulatory activity by Tregs (Tan et al., 2020). Among COVID-19 patients, Treg numbers have been significantly altered. Researchers found that both mild and severe COVID-19 patients had reduced levels of expression of CD4+ CD25+ CD127low Tregs and CD45RA+ Tregs. COVID-19 patients with severe disease had a much lower proportion of CD45RA+ Tregs than those with moderate disease, while moderate and severe COVID-19 patients with comparable numbers of CD45RO+ Tregs (Chen et al., 2020). There may be a connection between loss of CD45RA+ Tregs and increased levels of IL-10 in severe COVID-19 patients, which may result in increasing mortality rates in these patients (Wang et al., 2020). According to a separate study, Treg levels were notably lower in COVID-19 patients with severe cases, particularly those with severe symptoms (Qin et al., 2020). A study by Julika et al. also revealed that IL-10-producing Treg levels were particularly high in severe COVID-19 patients; the study also proved that Treg levels were elevated in mild COVID-19 patients as well. The severity of the disease may be linked to the production of IL-10 in certain tissues, such as the lungs (Neumann et al., 2020). In a report, Yang et al. described a patient with asymptomatic SARS-CoV-2 infection with CD3+CD8-CD4+CD127-CD25+ Treg surface phenotypes, a higher level of Tregs was present at day 7, a peak at day 22, and a downward trend at day 28 compared to healthy controls. During the early phase of asymptomatic infection, the proportion of incompetent T cells is high, suggesting that Tregs are inhibiting the activation and function of T cells (Impaired T cell functions along with elevated activated Tregs at the early stage of 2 asymptomatic SARS-CoV-2 infection). In some COVID-19 patients without Foxp3, the levels of CD45RA+CCR7+ Tregs in some COVID-19 patients decreased, while the levels of activated CD45RA-CCR7+ Tregs increased (De Biasi et al., 2020). The T cells of patients with COVID-19 are highly activated, Foxp3 expression is inhibited, and hyperactive CD25+ Tregs proliferate quickly and die before they become fully functional Tregs. Patients with COVID-19 may suffer from an overactive immune system and lung injury due to a reduction of Treg levels. The high frequency of hyperactivated CD25+ T cells may cause immunothrombosis, another symptom of COVID-19 severe manifestations (Kalfaoglu et al., 2020; Stephen-Victor et al., 2020; McGonagle et al., 2020). Another study found that severe COVID-19 patients had a higher number of Tregs with higher levels of FoxP3 expression than mild, recovered, or healthy controls. Furthermore, there is a higher expression of PD-1 and T-bet on Tregs in severe COVID-19 patients. Additionally, the researchers found that IL-16 and IL-8 induce the Treg phenotype in COVID-19 patients (Galvan-Pena et al., 2021). A recent study showed that CD4+ Foxp3+ regulatory T cells activate dendritic cells to produce antigen-specific immunity against emerging SARS-CoV-2 antigens (Uraki et al., 2021). In light of the changes in Treg factors and their potential role in the treatment of patients with COVID-19, Tregs will likely become a novel target for the treatment of COVID-19, which should be carefully examined in further experiments and clinical trials.

Th17 in COVID-19: Th17 is an IL-17-producing CD4+ T-cell subsets, which can be recognized by their production of interleukin-17 (IL-17) (Saini et al., 2022). IL-17 is a potent cytokine strongly associated with inflammatory processes and exhibits robust effects on stromal cells throughout the body (Tesmer et al., 2008). Research has shown that Th17 cells play a critical role in defending against extracellular bacteria, fungi, and autoimmune diseases by producing IL-17 (Yasuda et al., 2019).. Activated antigen-presenting cells such as dendritic cells, which secrete IL-23 as a result of taking up and processing pathogens, activate Th17 cells. There is evidence that many pro-inflammatory cytokines and chemoattractant are elevated in COVID-19 patients, including IL-1β, IL-6, IL17, TNF-α, GM-CSF, and IFN-γ. These pro-inflammatory cytokines and chemoattractant are related to Th17-mediated responses (Wu and Yang, 2020). A pro-inflammatory cytokine up-regulation during inflammation leads to polarization of Th17 cells (Pourgholaminejad et al., 2016).

COVID-19 patients with severe complications have also been reported to have higher numbers of proinflammatory Th17 cells in peripheral blood; the proinflammatory cytokine IL-17 may be a potential immunologically plausible modifiable target that could prevent ARDS, although more research is needed to support this (de Candia et al., 2021). In response to upregulation of Th17 cytokines, especially IL-17 and IL-22, mucin, fibrinogen and serum amyloid A are produced. There is a possibility that oedema formation in the mucosa of the respiratory tracts of COVID-19 patients is caused by systemic inflammation caused by Th17 (Wu and Yang, 2020). Apart from the proinflammatory cytokines, IL-6 also affects T cell polarization by stimulating the Th17 pathway and inhibiting TGF-β induced Treg production (Kimura and Kishimoto, 2010). A study by Hou et al. revealed that excessive IL-6 level promotes the formation of Th17 cells, and the IL-6 and IL-17 released from the transformed Th17 cells promote viral persistence by inhibiting apoptosis of virus-infected cells (Hou et al., 2014). In the case of influenza virus infection, increased neutrophils are found in the airspace following IL-17 production, which contributes to acute lung injury with a high mortality rate (Crowe et al., 2009). In several human diseases, recent research showed that IL-17 functions were important for suppressing viral infections and minimizing tissue pathology in different settings of viral infection (Ma et al., 2019). A study suggested that COVID-19 patient with pneumonia produced more *in vitro* IL-17, which boosted the inflammation response and activated neutrophils *in vitro* CD4+ and CD8+ T cells produced more of IL-17 in patients with pneumonia. Furthermore, peripheral blood cells from patients expressed less CCR6 and more CD161, both of which are characteristic of TH17 and mucosal associated invariant T (MAIT) cells, respectively. A higher IL-17 production was observed in lung cells compared to peripheral blood cells. Overall, these findings support the importance of IL-17 in COVID-19 and suggest that biological drugs, which are already available, might be useful in developing novel therapeutic approaches (De Biasi et al., 2020).. MERS-CoV patients also showed elevated levels of proinflammatory molecules, including IFN-γ, TNF-α, IL-15, and IL-17, indicating a Th1/Th17 response (Mahallawi et al., 2018). COVID-19 is heavily influenced by IL-17, which recruits and activates neutrophils, which migrate to the lungs and participate in pathogenesis. They demonstrate that activation of T cells in COVID-19 patients is significantly skewered towards Th17 functional phenotype (De Biasi et al., 2020). It is possible that severe cases of COVID-19 will cause a drastic autoimmune response due to the lower proportion of Tregs. In COVID-19 patients, it has been suggested that the over-representation of Th17/Treg may affect the levels of regulatory cytokines (IL-4, IL-10, TGF-β, and IL-35) and the ability to tolerate self-antigens, ultimately leading to autoimmune inflammation (Qin et al., 2020). Huang et al. also observe an increase in IL-17 in COVID-19 patients that is more prominent in intensive care compared to non-intensive care and controls (Huang et al., 2020), and Zumla et al. postulate that blocking IL-17 could improve respiratory distress syndrome-related mortality and apparent aberrant immune response in COVID-19 patients (Zumla et al., 2020). Though blocking IL-17 may decrease Th1 mediated inflammation, SARS-CoV-2 also appears to increase Th2 cytokine production (IL-4 and IL-10) that suppress Th1/Th17 mediated inflammation (Huang et al., 2020). In light of these findings, further investigation into the mechanisms underlying the action of IL-17 as a possible COVID19 therapeutic target is essential.

NKT cells in COVID-19: Natural killer T (NKT) is a heterogenous group of non-conventional T cells. In addition to having the properties of both T cells and natural killer cells, NKT cells also share aspects of both categories. This group of cells recognizes the non-polymorphic CD1d molecule, which presents antigens by binding to lipids and glycolipids of both self and foreign origin. T cells in peripheral blood make up only about 1% of the total T cells (Godfrey et al., 2004). There are two types of natural killer T cells (NKTs): type I and type II NKTs. Type I NKT cells are more commonly called invariant NKT (iNKT) and it recognizes glycolipid antigens contained within CD1d molecules (Pellicci et al., 2020). Additionally, proinflammatory cytokines produced during an infection can activate iNKT cells (Slauenwhite and Johnston, 2015). An NKT cell that responds to lipid antigens belongs to type II. It is CD1d-restricted. Researchers have found that type II NKT cells are able to recognize hydrophobic peptides presented on CD1d as well as lipid antigens. They are a distinct population of cells with immune-regulatory capabilities that circulate and reside in tissues. Evidence suggests they play a role in regulating immunity to pathogens/tumors, as well as in autoimmune and metabolic diseases (Dhodapkar and Kumar, 2017; Singh et al., 2018).

A study has shown that in mild or moderate cases of COVID-19, the number of CD160+ NKT cells increases, which is associated with a quicker resolution of the infection by direct cytotoxicity. There was also significant enrichment for the *FCGR3A* gene in this cluster, which suggests that it could play a role in antibody-dependent cytotoxicity. In severe cases of COVID-19, the CD160 cluster was noticeable absent (Zhang et al., 2020). There has been evidence that circulating iNKT cells are activated by IL-18 in severe cases of COVID-19 (Jouan et al., 2020), IL-18 cytokine is associated with T cell activation during viral infections (Tsai et al., 2015). iNKT cells are also seen to be declining along with IFN-γ production by iNKT cells in severe cases of COVID-19. iNKT cells from patients in intensive care units displayed increased CD69 and PD-1 expression at day 15, but PD-1 was still highly expressed on iNKT cells (Jouan et al., 2020). Interestingly, another study found reduced levels of NKT (CD3+ CD56+) cells in patients with severe disease (Zingaropoli et al., 2021), but this finding is inconsistent with those from other studies (Zhang et al., 2020; Mazzoni et al., 2020). It is fascinating that CD3+CD56+ T cells are associated with COVID-19 severity, even though they are immensely unlikely to be CD1d-restricted NKT cells (Kreutmair et al., 2021). The CD3+CD56+ T cells are heterogeneous, with a variety of non-MHC-restricted nonconventional T cells such as mucosal associated invariant T (MAIT) cells, as well as γδ T cells (Godfrey et al., 2015). CD56-expressing cells may also include T cells associated with cytotoxicity, like CD8+ T cells (Van Acker et al., 2017). A deeper analysis of this population may aid in understanding how the immune system responds to severe COVID-19.

**Figure 3:**
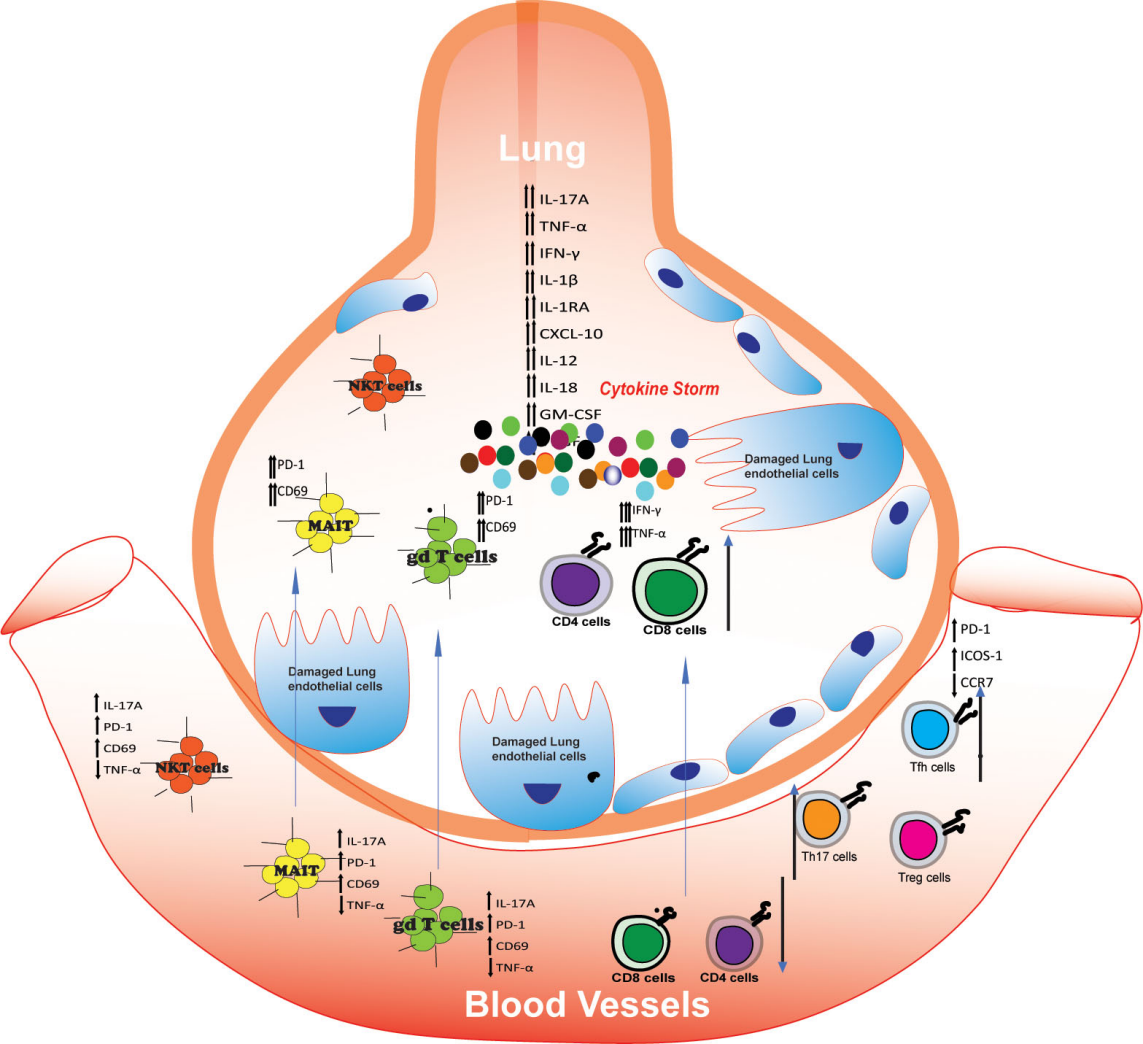
T cells in severe COVID-19: presence and activation.: Numbers of CD4 and CD8 decreased in peripheral blood (lymphopenia) while activated CD4 and CD8 cells are increased in lung secernated excessive amount of TNF-α and IFN-γ which damaged lung and inhibits function of Tregs. IL-17 secreting Th17 cells and follicular helper T (Tfh) cells increased in peripheral blood of COVID-19 patients, however there are conflicting finding in case of Tregs. The number of NKT, MAIT, and γδ T cells decreases in peripheral blood and is detected in lung aspirates with heightened levels of activation, as evaluated by CD69 and PD-1. Activated conventional and unconventional T cells in the lung may secrete multiple inflammatory cytokines such as IL-17A, IL-6, TNF-α, and IFN-γ that contribute to the cytokine milieu and/or disease pathology.

γδ T cells in COVID-19: γδ T cells are engaged in the adaptive immune response as well as the early immunological response to infections or malignant transformation (Saini et al., 2018). Despite accounting for approximately 0.5-5 percent of circulating T cells in homeostatic settings, γδ T cells are potent effector cells (Tarique et al., 2017).Research into the involvement of T cells so far has largely focused on T cells. Thus, a limited amount of information is available regarding the involvement of γδ T cells. It’s important to note first and foremost that the lymphopenia being reported affects αβT cells as much as γδ T cells (Lei et al., 2020; Odak et al., 2020). An interesting finding was that the patients with the most severe symptoms had the least amount of T cells in their body (Rijkers et al., 2020). The question arises, then, whether the number of γδ T cells in patients is a good indicator of disease severity. Furthermore, there appears to be a significant change in the phenotype of γδ T cells in patients. These changes are the subject of conflicting reports. In one study, both percentages and absolute numbers indicate a shift towards nave behavior (Odak et al., 2020). Over the course of two weeks, in contrast, a shift towards an effector phenotype was observed in a different study with significantly less patients. There is evidence that a lymphopenia has been observed, similar to what has been described in γδ T cells, along with an overall reduction of the naive phenotype. COVID 19 patients’ increased activation marker CD25 also supports this conclusion (Carter et al., 2020). In addition, CD69 and PD-1 are highly expressed on γδ T-cells, indicating strong evidence that the immune system is working (Chen and John Wherry, 2020). Both the frequency and absolute number of naive (CD45RA+CD62L+) γδ T cells were elevated in blood in mild and severe cases of COVID-19, whereas the number of effector (CD45RA-CD62L+) γδ T cells decreased proportionately. Several researchers hypothesized that effector-like γδ T cells could be recruited to the lungs of COVID-19 patients, where they would help combat the infection (Odak et al., 2020). In order to better understand γδ T cell and COVID-19 relationships, researchers need to investigate the cytokine release syndrome (CRS), often called cytokine storm and fibrosis, two of the most serious complications suffered by COVID-19 patients. A number of cytokines are produced and secreted by γδ T cells, including IFN-γ, TNF-α, IL-6, and IL-17 (**Figure 3**). Besides interacting with other immune cells, they also perform cytolysis using cytotoxic perforin and granzyme (Battistini et al., 2005). Moreover, γδ T cells are capable of defending against viral infection by secreting IFN-g, up-regulating NKG2D, and producing perforin, granzyme B, and FasL (Xue et al., 2017). The gd cells display increased activation and preferentially produce IL-17A while avoiding producing IFN-γ (Rijkers et al., 2020). Researchers found that endotracheal aspirates contain higher concentrations of inflammatory cytokines like IL1β, IL-6, and IFN-γ than matched blood samples. They also found the presence of γδ T cells with heightened levels of activation in COVID-19 sample aspirates (Jouan et al., 2020). There are strong indications that γδ T cells play an important role in the immune response of COVID-19 in the most resistant population. There is no doubt that γδ T cells have great potential in helping the fight against the SARS-Cov-2 pandemic, but further research is needed.

MR1-restricted mucosal-associated invariant T (MAIT) cells in COVID-19: The major histocompatibility complex class I-related molecule (MR1) recognizes vitamin B metabolites from microbes at mucosal sites and plays a putative role in antimicrobial immunity (Treiner et al., 2003). The number of MAIT cells varies widely among healthy individuals, with up to half of all T cells in the liver and 2 to 5% of T cells in the blood containing MAIT cells (Godfrey et al., 2015). MAIT cells are frequently found in low numbers in patients with viral infections (Leeansyah et al., 2013; Barathan et al., 2016; Paquin-Proulx et al., 2017). The cytokines IL-12 and IL-18 have been shown to activate MAIT cells in a TCR-independent manner, though they are not directly activated by viruses (Ussher et al., 2014). Proinflammatory cytokines such as IFN-γ, TNF-α, and IL-17 are rapidly produced by activated MAIT cells (van Wilgenburg et al., 2016).

There is evidence that MAIT cells can be activated by IL-18 in a non-receptor-dependent manner during SARS-CoV-2 infection, since CD69 expression on MAIT cells correlates with high plasma IL-18 levels (Jouan et al., 2020). A decrease in circulating MAIT cells was observed during mild and moderate infections, which is thought to reflect the recruitment of these cells into the airways and their activation-induced death (Flament et al., 2021). Despite the underlying causes of COVID-19 and regardless of the patient’s course of disease, MAIT cells were highly activated and produced large amounts of proinflammatory cytokines such as IL-17A and TNF-a. The cytokines IL-17A and TNF-α, as well as granzyme B and perforin were not upregulated in response to MAIT cell-specific *in vitro* stimulation. Researchers have observed altered MAIT cell cytokine expression profiles in COVID-19, as well as disruptions in their anti-bacterial and antiviral function and thus contributed to the understanding of the COVID-19 immunopathogenesis (Deschler et al., 2021). Among COVID-19 patients, a higher frequency of MAIT cells was observed in endotracheal aspirates than matched blood samples (Jouan et al., 2020). There was also an inverse correlation between the frequency of MAIT cells and the level of IL-17C (pro-inflammatory cytokine) in plasma of COVID-19 patients. SARS-CoV-2 infections were associated with both MAIT cell recruitment and increased lung epithelial inflammation, supporting the possible link between these features (Parrot et al., 2020). A report suggested that MAIT cells also expressed CXCR3 (Saha et al., 2013), several studies have shown an increase in expression of early activation marker CD69 on MAIT cells and a decrease in expression of the homing marker CXCR3 in mild and severe cases of COVID-19 (Parrot et al., 2020; Flament et al., 2021; Deschler et al., 2021). As opposed to conventional CD4+ and CD8+ T cells, MAIT cells were characterized by higher levels of CD69 expression and lower levels of CXCR3, and CXCR3 expression on MAIT cells was inversely correlated with CD69 expression. In the airways, CD69+CXCR3- MAIT cells replicated this circulating phenotype, which was found to be the major source of IL-17A (Parrot et al., 2020). Both mild and severe COVID-19 cases have been associated with higher levels of IL-17A, with the highest levels observed in severe cases. IL-17 activates inflammatory pathways that can cause tissue damage and aggravate disease; therefore, it is likely to contribute to the development of COVID-19 (Fadlallah et al., 2021). There were reduced frequency and an activated phenotype of circulating MAIT cells in COVID-19 patients regardless of disease severity, as demonstrated by high levels of IL-17A, TNF-α, CD38, CD69, and HLA-DR expression (Deschler et al., 2021).

These results imply a pathogenic role for MAIT cells in COVID-19 patients, with a reduced number of cells in the periphery and increased numbers in the lung and an earlier active phenotype correlated with a poorer outcome. The activation of MAIT cells has also been associated with vaccine immunity as MAIT cells boost CD8 T cell response to adenoviral vaccines. The MAIT cell effector responses may have different impacts depending on the disease stage, but more research is needed to dissect these differing contributions to antiviral immunity in the COVID-19 setting.

## Conclusion

SARS-CoV-2 infection triggers an inflammatory response that can lead to debilitating illness and cytokine storms, resulting in cytokine storm and debilitating health condition. The emerging studies into various conventional and non-conventional T cell subsets that are involved in both the pathogenesis and resolution of COVID-19 reveal interesting and intriguing information regarding how these cells are involved in both the pathogenesis and resolution of the disease. It remains unclear how the underlying mechanisms act and what stage-dependent functions they perform. A variety of T cell subsets have been shown to become activated in response to viral infections, including COVID-19, and to have antiviral capabilities, although it remains to be seen whether such functions can be successfully exploited for therapy. The loss or dysfunction of T cells or the overactivation of T cells may also increase the susceptibility to other microbial infections, as well as autoimmunity, which may result in serious complications, including sepsis, as well as understanding how SARS-CoV-2 is transmitted. To better understand T cell subsets, which are important for both innate and adaptive immunity, we need to learn more about their complex antimicrobial effector functions.

## Author contributions

Conceptualization, methodology, original draft preparation, article writing, visualization, review and editing by MT; software work, validation, data curation, review, and editing by HN, NM, MS, ST, TZ, AA, AH, HS, CS, and AS; resources, review and editing, super-vision, project administration, funding acquisition by MS and TZ. All authors have read and agreed to the published version of the manuscript.

## Funding

This research work was funded by the Institutional Fund Projects under grant no. (IFPDP-7-22). Therefore, the authors gratefully acknowledge technical and financial support from Ministry of Education and Deanship of Scientific Research (DSR), King Abdulaziz University (KAU), Jeddah, Saudi Arabia.

## Conflict of interest

The authors declare that the research was conducted in the absence of any commercial or financial relationships that could be construed as a potential conflict of interest.

## Publisher’s note

All claims expressed in this article are solely those of the authors and do not necessarily represent those of their affiliated organizations, or those of the publisher, the editors and the reviewers. Any product that may be evaluated in this article, or claim that may be made by its manufacturer, is not guaranteed or endorsed by the publisher.
